# Effects of Stems and Leaves of *Panax notoginseng* on mRNA Expression of TLR Signaling Pathway in Hepatic Tissue of Duzang Pigs

**DOI:** 10.3390/genes16070781

**Published:** 2025-06-30

**Authors:** Na Zhang, Lanlan Yi, Yuxiao Xie, Huijin Jia, Guangyao Song, Wenjie Cheng, Wenzhe Shi, Junhong Zhu, Sumei Zhao

**Affiliations:** College of Animal Science and Technology, Yunnan Agricultural University, Kunming 650201, China; 2023210409@stu.ynau.edu.cn (N.Z.); yilanlan0217@163.com (L.Y.); 2022110080@stu.ynau.edu.cn (Y.X.); 2022210413@stu.ynau.edu.cn (H.J.); songguangyao1990@163.com (G.S.); cwj210365@163.com (W.C.); 18287473985@163.com (W.S.); 2024022@ynau.edu.cn (J.Z.)

**Keywords:** Duzang pigs, Panax notoginseng stems and leaves, liver, Toll-like receptor, mRNA expression level

## Abstract

**Background/Objectives**: *Panax notoginseng* stems and leaves (PNSLs) have shown limited adoption as a forage component in commercial livestock operations due to low utilization rates. **Methods**: This study was designed to add 10% and 20% PNSLs in the diet of Duzang pigs. Hepatic tissues were collected to investigate the expression levels of Toll-like receptor (*TLR*), *MyD88*, *TRIF* genes, and downstream cytokines within the TLR signaling pathway. **Results**: 10% and 20% PNSLs in the diet significantly up-regulated mRNA expression levels of *TLR3*, *TLR6, TLR7*, *TLR8*, *TRIF*, *IL-10*, *IFN-α* and *IFN-β*, while they down-regulated *TLR5*, *TLR9*, *TNF-α* and *IL-1β* in Duzang pigs. No significant effects were observed on the expression of *TLR2* and *IL-6*. **Conclusions**: Different amounts of PNSLs modulated the TLR signaling pathway mRNA expression levels in the hepatic tissues of Duzang pigs.

## 1. Introduction

Yunnan, as the southwestern region of China, has unique natural conditions that have given birth to many excellent local pig breeds. Among them, the high-altitude (3500–4500 m) Diqing Tibetan Autonomous Prefecture has cultivated a plateau pig breed, the Tibetan pig in Diqing, which has strong disease resistance, stress resistance, and cold resistance, and can tolerate coarse feed [1]. The Duroc pig exhibits phenotypically distinctive traits including a broad thoracic frame, well-developed musculature, and structurally sound limbs, these characteristics collectively represent lean-type swine breeds [2]. Compared with the Duroc pig, the Tibetan pig in Diqing exhibits smaller body conformation and slower growth, but its stress resistance and disease resistance are stronger, and it is suitable for survival under harsh environmental conditions. The Duzang pig is a hybrid breed of the Duroc pig and Tibetan pig in Diqing. Through improvement, the Duzang pig exhibits enhanced adaptability to high-altitude environments, has superior meat quality and robust disease resistance. *Panax notoginseng* (PN), a medicinal plant endemic to Yunnan Province, plays significant roles in anti-inflammatory therapies and the treatment of diabetes, cancer, metabolic, and neoplastic diseases [3,4]. However, its stems and leaves are often discarded as by-products without effective utilization.

As a important immune-related organ [5], the liver significantly contributes to innate immunity through its specialized cellular architecture—such as its Kupffer cells, hepatocytes, and hepatic stellate cells—both detecting phagocytose and eliminating bacterial and viral pathogens, while triggering [6] and orchestrating immune responses [5]. Toll-like receptors (TLRs), a conserved family of pattern recognition receptors, serve as the primary sentinels in initiating innate immune responses [7]. TLRs are localized either on the plasma membrane (TLR1, TLR2, TLR4, TLR5, TLR6) or within intracellular compartments (TLR3, TLR7, TLR8, TLR9) [8], all of which can transduce signals through both MyD88-dependent and Toll/interleukin-1 receptor domain (TIR)-containing adapter-inducing interferon-β-dependent pathways [9].

MyD88 and TRIF are critical adaptor proteins in TLR signaling cascades, each mediating distinct downstream immune responses. The bridging of inflammatory signaling can be achieved through TLR-mediated MyD88 protein. MyD88 exerts its biological functions by means of kinase cascade activation [10]. TRIF plays an indispensable role in regulating TLRs-mediated necroptosis signaling pathways [11]. As essential intercellular communicators, cytokines orchestrate inflammation promoting and suppressing processes after sensing upstream effector proteins, thereby maintaining immune homeostasis [12], including but not limited to interleukin, tumor necrosis factor, and interferon.

Basal diets were supplemented with 10% and 20% PNSLs in this study. Bioinformatics approaches were employed to predict protein structures and compositions of TLRs, while real-time quantitative PCR (qPCR) was utilized to determine mRNA expression levels of TLRs, MyD88, TRIF and downstream cytokines. This study aims to establish the optimal PNSLs percentage of Duzang pig diets and provide fundamental data.

## 2. Materials and Methods

### 2.1. Treatment of the PNSLs

The collection and processing of PNSLs are presented in our previous research [13,14]. Fresh PNSLs were harvested from Yanshan County (Wenshan, Yunnan Province, China), and the proximate analysis was conducted (Table 1).

### 2.2. Animal Management

All animal experiments were approved by the Animal Ethics Committee of Yunnan Agricultural University. Our previous research had details of animal experiments [13,14]. Thirty healthy Duzang pigs (Duroc pig♂ × Tibetan pigs in Diqing♀) with normal feeding intake, a good mental condition, and similar body weights (75.84 ± 7.43 kg) [13,14] were selected from Yanshan County, Wenshan Prefecture, Yunnan Province. Pigs were randomly assigned to three dietary treatment groups (*n* = 10/group). The feed quality and nutritional level of the three groups were good. The control group (CG) received a corn-soybean meal basal diet, while experimental groups I (EG I) and II (EG II) were fed the same basal diet supplemented with 10% and 20% Panax notoginseng stems and leaves, respectively (Supplementary Table S1). The experimental period lasted for 32 days. During the period, each Duzang pig could freely eat the diet and drink water, and was kept separately in a pen. Regular manure removal and disinfection were performed to maintain hygienic housing conditions.

### 2.3. Gene Sequence Analysis

The complete gene sequences were obtained from a public database, and the protein domains were predicted using SMART (http://smart.embl-heidelberg.de/ accessed on 1 May 2023). Using EXPASY (http://web.expasy.org/protparam/ accessed on 1 May 2023), analysis of TLR protein amino acid composition, molecular weight (mw) and the theoretical value of theoretical isoelectric points (pI) was conducted.

### 2.4. Extraction of RNA

Hepatic tissues were aseptically collected from five randomly selected Duzang pigs per treatment group. Tissue samples were dissected into fragments, immediately transferred to pre-chilled 1.5 mL cryotubes, flash-frozen in liquid nitrogen, and stored at −80 °C until analysis. Approximately 40 mg of hepatic tissue was aseptically excised using autoclaved scissors, minced into fine fragments, and transferred to a liquid nitrogen-prechilled mortar for grinding into a homogeneous powder. The powdered tissue was then aliquoted into a 1.5 mL microcentrifuge tube, mixed with 1 mL of Trizol reagent, vortexed thoroughly and incubated at room temperature for 5 min. A volume of 0.2 mL chloroform was added, followed by vortexing for 15 s and incubation for 3 min at room temperature. Centrifugation was performed at 12,000× *g* for 15 min at 4 °C, and the aqueous phase was collected. The aqueous phase (0.5 mL) was carefully transferred to a fresh 1.5 mL microcentrifuge tube. An equal volume of isopropanol (0.5 mL) was added, and the solution was gently inverted 8–10 times to ensure complete mixing. After incubation at room temperature for 10 min, the mixed solution was centrifugated at 12,000× *g* for 10 min at 4 °C. After centrifugating, a gel-like RNA pellet was visible at the bottom and sidewalls of the tube. The supernatant was carefully poured out to avoid the sediment flowing out with the liquid. The RNA was washed with 1 mL of 75% ethanol (3:1, ethanol and DEPC-H_2_O) by vortexing, followed by centrifugation at 7500× *g* for 5 min at 4 °C. The supernatant was discarded, and the tube was air-dried at room temperature for 1–3 min to evaporate residual ethanol. The RNA pellet was then dissolved in 20–30 μL of DEPC-H_2_O by gentle pipetting.

### 2.5. RNA Detection

RNA concentration and OD_260/280_ values were determined with a DU640 spectrophotometer (MIULAB, Hangzhou, China). Total RNA isolated from Duzang pig hepatic tissues was analyzed by agarose gel electrophoresis to evaluate integrity and detect potential degradation or contamination.

### 2.6. Reverse Transcription

Total RNA was reverse transcribed into cDNA using the two-step reverse transcription procedure according to the manufacturer’s instructions of the TaKaRa kit (Fengke Biotechnology Co., Ltd., Kunming, China). The genomic DNA removal reaction mixture was first incubated at 42 °C for 2 min and then stored at 4 °C. Subsequently, the reverse transcription reaction system was added and subjected to the following process: 37 °C for 15 min, 85 °C for 5 s, followed by storage at 4 °C.

### 2.7. Primer Design

All primers were synthesized (Qingke Biotechnology Co., Ltd., Kunming, China) with sequences listed in Supplementary Table S2, including Toll-like receptors (TLR1-TLR9), pivotal proteins of TLR signaling pathway (MyD88, TRIF), downstream cytokines (IL-10, IL-lβ, IL-6, TNF-α, IFN-α, IFN-β) and reference gene (β-actin).

### 2.8. qPCR

The qPCR was performed according to the manufacturer’s instructions of the TaKaRa Tli RNaseH Plus kit (Fengke, Kunming, China). qPCR reactions were carried out using the SYBR Green staining method. The total reaction volume was 20 μL, with all procedures conducted on ice. The following quantities were used: TB GreenPremix Ex Taq I—10 μL, PCR Forward Primer—0.4 μL, PCR Reverse Primer—0.4 μL, cDNA obtained through reverse transcription—1 μL, RNase-Free ddH_2_O— 8.2 μL. qPCR reaction procedure consists of 1 cycle at 95 °C for 3 min for pre-denaturation, 40 cycles at 95 °C for 15 s for denaturation, 30 s (the temperature is shown in Supplementary Table S2) for annealing, fluorescence collection, detection, and 30 s for extension at 72 °C. The relevant data were obtained using the qPCR instrument (Bio-rad, Herculaneum, Hercules, CA, USA).

### 2.9. Data Processing

mRNA expression quantification was performed using qPCR with β-actin serving as the reference gene. After obtaining the threshold cycle (Ct) values for both target genes and the reference gene in triplicate measurements, relative mRNA expression levels were normalized and calculated according to the 2^−ΔΔCt^ method. The specific calculation formula is shown as follows.

Ratio = 2^−∆Ct Target(Sample − Calibrator)^/2^−∆Ct18s(Sample − Calibrator)^

Sample represents the experimental group, Calibrator the control group. Ct represents the PCR cycle number at which the fluorescence signal exceeds a predetermined threshold.

The experimental data were preprocessed using Excel 2016. Statistical analyses were performed using SPSS 22.0, including one-way ANOVA followed by Duncan’s multiple range test for comparing (significance level set at *p* < 0.05, extremely significance level set at *p* < 0.01). Additionally, data visualization was conducted with GraphPad Prism 8. Spearman’s rank correlation analysis was employed to assess the correlative expression patterns of genes in the TLR signaling pathway.

## 3. Results

### 3.1. Amino Acid Composition and Protein Structure Prediction of TLRs

The amino acid composition of TLR proteins were analyzed using the ProtParam (online, 2023) tool on the EXPASY bioinformatics platform (Supplementary Table S3). The molecular weights and theoretical isoelectric points are shown in the following Table 2.

**Figure 1:**
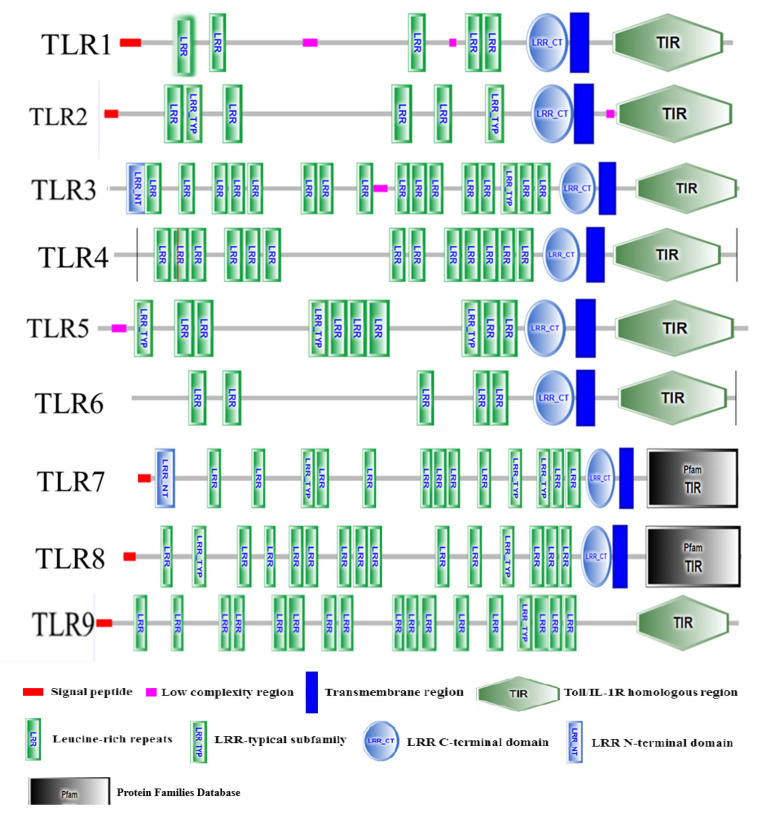
Sketch of SMART predicted protein structural domains of porcine TLRs.

Protein domain prediction using SMART software (online, 2023) revealed that porcine TLRs (TLR1-9) all contain TIR domains. Specifically, TLR1/2/3/4/5/6/7/8 possess complete LRR motifs, LRR-CT motifs, transmembrane domains, and TIR domains, whereas TLR9 lacks both the LRR-CT and transmembrane domains (Figure 1).

### 3.2. Effects of PNSLs on Hepatic TLR Signaling Pathway mRNA Expression Levels of Duzang Pigs

#### 3.2.1. Total RNA Extraction from Hepatic Tissue

Electrophoretic analysis of hepatic total RNA from Duzang pigs revealed three sharp ribosomal RNA bands (28S, 18S and 5S). The bands indicated well-preserved RNA integrity.

Quantification analysis showed that the total RNA extracted from Duzang pig hepatic tissues had concentrations ranging from 849 to 1258 ng/μL with OD_260/280_ of 1.8–2.1. A high purity is suitable for downstream applications including reverse transcription and qPCR.

#### 3.2.2. Effects of PNSLs on TLRs mRNA Expression Levels

**Figure 2:**
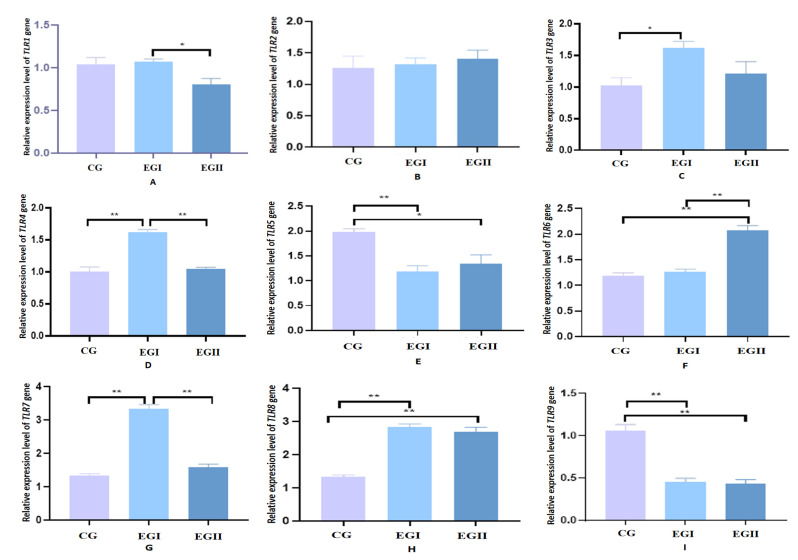
Relative mRNA expression levels of TLRs in hepatic tissues of Duzang pigs. (**A**) Relative expression level of TLR1. (**B**) Relative expression level of TLR2. (**C**) Relative expression level of TLR3. (**D**) Relative expression level of TLR4. (**E**) Relative expression level of TLR5. (**F**) Relative expression level of TLR6. (**G**) Relative expression level of TLR7. (**H**) Relative expression level of TLR8. (**I**) Relative expression level of TLR9 *n* = 5. CG represents the control group, EG I represents the experimental groups I, and EG II represents experimental groups II. **, at the 0.01 level, highly significant. *, at the 0.05 level, significant.

*LR5* and *TLR9* showed significantly higher expression in the CG compared to EG I and EG II. *TLR3*, *TLR4*, *TLR7* and *TLR8* were significantly upregulated in EG I versus CG. EG I exhibited significantly elevated expression of *TLR1*, *TLR4* and *TLR7* relative to EG II. *TLR6* expression was markedly higher in EG II than in both EG I and CG (Figure 2).

#### 3.2.3. Effects of PNSLs on mRNA Expression Levels of Pivotal Proteins in TLR Signaling Pathway

**Figure 3:**
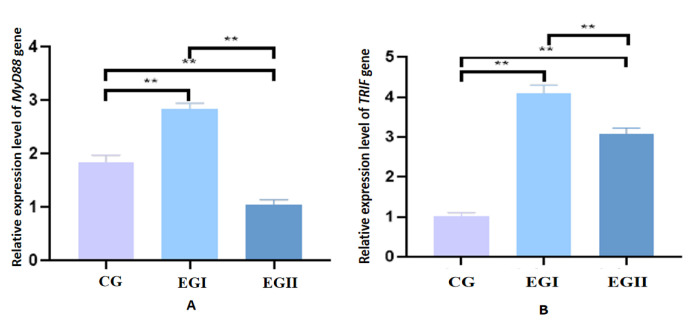
Relative mRNA expression levels of pivotal proteins in hepatic tissues of Duzang pigs (**A**). Relative mRNA expression level of *MyD88.* (**B**) Relative mRNA expression level of *TRIF n* = 5. CG represents the control group, EG I represents the experimental groups I, and EG II represents experimental groups II. **, at the 0.01 level, highly significant.

The expression levels of both *MyD8*8 and *TRIF* were significantly elevated in EG I compared with CG and EG II. Notably, CG exhibited higher *MyD88* expression than EG II, whereas *TRIF* expression was significantly greater in EG II than in CG (Figure 3).

#### 3.2.4. Effects of PNSL on Downstream Cytokines mRNA Expression Levels in TLR Signaling Pathway

**Figure 4:**
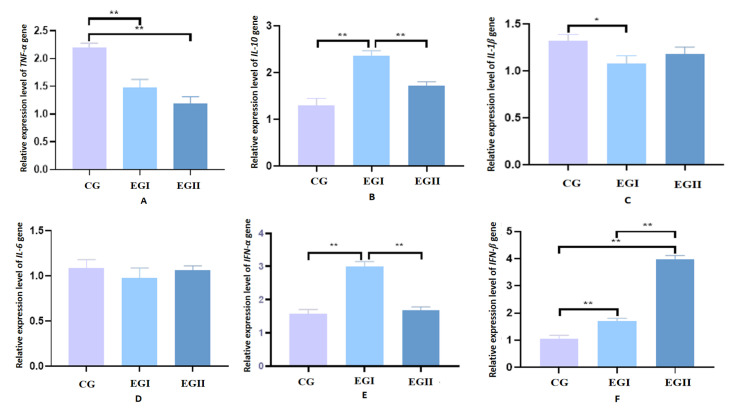
Relative mRNA expression levels of cytokines in hepatic tissues of Duzang pigs. (**A**) Relative mRNA expression level of *TNF-α.* (**B**) Relative mRNA expression level of *IL-10.* (**C**) Relative mRNA expression level of *IL-1β.* (**D**) Relative mRNA expression level of *IL-6.* (**E**) Relative mRNA expression level of *IFN-α.* (**F**) Relative mRNA expression level of *IFN-β n* = 5. CG represents the control group, EG I represents the experimental groups I, and EG II represents experimental groups II. **, at the 0.01 level, highly significant. *, at the 0.05 level, significant.

The expression levels of *TNF-α* and *IL-1β* were significantly up-regulated in the CG compared with the EG I. Conversely, EG I demonstrated significantly higher mRNA expression of *IL-10* and *IFN-α* than both CG and EG II. Notably, *IFN-β* expression in EG II was markedly elevated relative to the CG and EG I. However, no statistically significant differences were observed in *IL-6* expression among CG, EG I and EG II (Figure 4).

### 3.3. Principal Components and Correlation Analysis of TLR Signaling Pathway Expression

#### 3.3.1. Cluster Analysis and Principal Component Analysis

**Figure 5:**
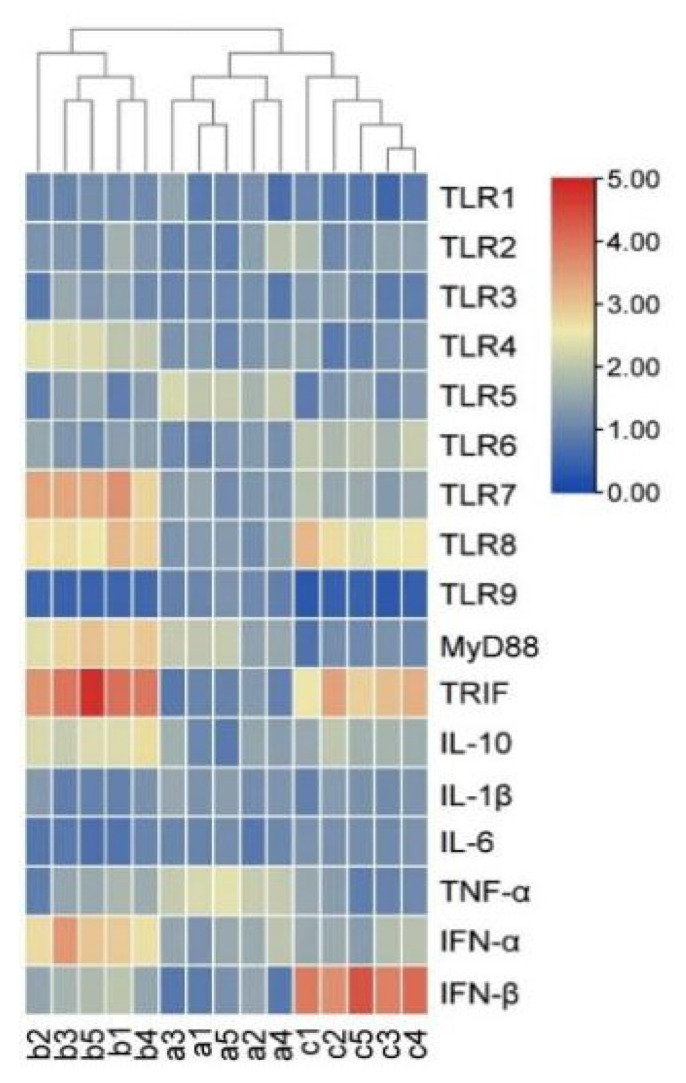
Clustering map of mRNA relative expression. Note: a1, a2, a3, a4, a5 shows CG. b1, b2, b3, b4, b5 shows EG I. c1, c2, c3, c4, c5 shows EG II. As the color bar changes from 0.00 to 5.00, it shifts from blue to red, indicating that the mRNA expression levels of the gene vary from low to high in different samples of different groups.

Hierarchical clustering analysis (heatmap) was performed to visualize the expression patterns of TLR signaling pathway-related genes in the hepatic tissues of CG, EG I and EG II (Figure 5). The color gradient from red to blue indicates high to low relative expression levels, respectively. Notably, substantial disparities in expression levels abundance were observed between EG I, EG II and CG.

**Figure 6:**
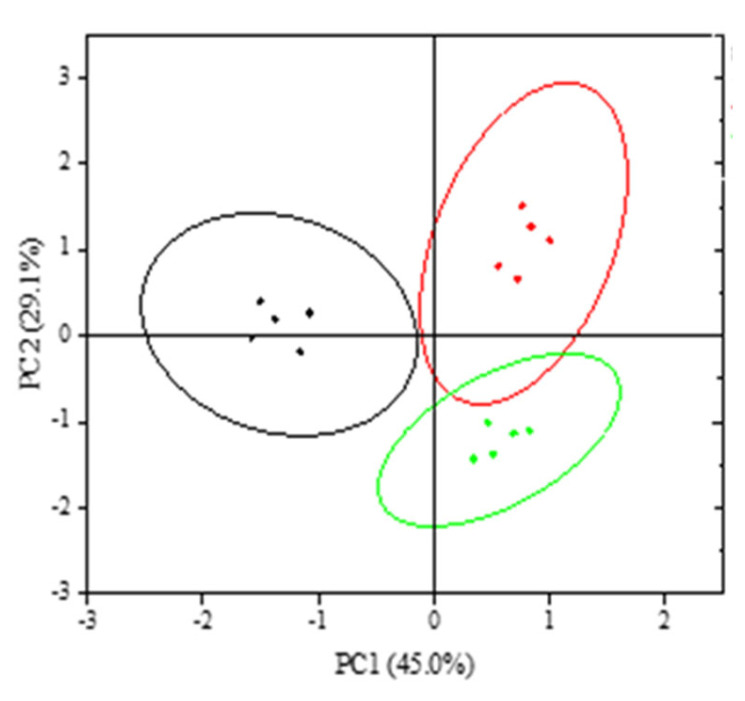
Principal component analysis Note: black represents the CG, red represents EG I, and green represents EG II.

Principal component analysis (PCA) was conducted to assess the differential expression of TLR signaling pathway genes. PC1 accounted for 45.0% of the variance, while PC2 explained 29.1% (Figure 6). Notably, experimental group samples exhibited closer clustering patterns.

#### 3.3.2. Correlation Analysis of the mRNA Expression Levels of TLRs and Pivotal Proteins

*MyD88* was extremely significantly correlated with the relative mRNA expression levels of *TLR6* (*p* < 0.01) and *TLR9* (*p* < 0.01), significantly correlated with *TLR1* (*p* < 0.05), negatively correlated with *TLR2*, *TLR6* (*p* < 0.01), *TLR7*, *TLR8*, and positively correlated with *TLR1* (*p* < 0.05), *TLR4*, *TLR5, TLR9*.

*TRIF* was extremely significant correlated with the relative mRNA expression level of *TLR7* (*p* < 0.01) and *TLR8* (*p* < 0.01), significantly correlated with *TLR4* (*p* < 0.05), *TLR5* (*p* < 0.05), positively correlated with *TLR1*, *TLR2*, *TLR3, TLR4*, *TLR6*, *TLR*7, *TLR* 8, negatively correlated with *TLR5* (*p* < 0.05), *TLR9*, *MyD88* (Table 3).

#### 3.3.3. Correlation Analysis Between TLRs and Cytokine mRNA Expression Levels

*TLRs* and cytokines were positively or negatively correlated in different degrees (Supplementary Table S4).

*IL-10* was extremely significant correlated with the relative mRNA expression level of *TLR7* (*p* < 0.01) and *TLR8* (*p* < 0.01), significantly correlated to *TLR5* (*p* < 0.05), and negatively correlated to *TLR5* (*p* < 0.05), *TLR9*. *IL-10* was positively correlated with *TLR1*, *TLR2*, *TLR3*, *TLR4*, *TLR6*, *TLR7* (*p* < 0.01), and *TLR8* (*p* < 0.01).

*IL-1β* as opposed to *TLR3* (*p* < 0.05) mRNA expression was significantly related. *IL-1β* was negatively correlated with *TLR1*, *TLR2*, *TLR3* (*p* < 0.05), *TLR4*, *TLR6*, *TLR7*, *TLR8*, *IL-10*; the levels of *TLR5*, *TLR9* were positively correlated with *IL-1β*.

*IL-6* was negatively correlated with *TLR1*, *TLR2, TLR4*, *TLR5*, *TLR7*, *TLR9*, and *IL-10* mRNA expression; *IL-6* was positively correlated with *TLR3*, *TLR6*, *TLR8*, *IL-1β*.

*TNF-α* was extremely significant correlated *TLR6* (*p* < 0.01) and *TLR9* (*p* < 0.01) mRNA relative expression, significantly correlated to *TLR5* (*p* < 0.05); *TNF-α* and *TLR2*, *TLR4*, *TLR6* (*p* < 0.01), *TLR7*, *TLR8*, *IL-10* showed a negative correlation; *TNF-α* and *TLR1*, *TLR3*, *TLR5* (*p* < 0.05), *TLR9* (*p* < 0.01), *IL -1β*, and *IL-6* were positively correlated.

*IFN-α* was extremely significantly correlated with the relative mRNA expression level of *TLR4* (*p* < 0.01) and *IL-10* (*p* < 0.01), significantly correlated with *TLR7* (*p* < 0.05) and *TLR8* (*p* < 0.05). *IFN-α* was negatively correlated with *TLR5*, *TLR6*, *TLR9*, *IL-1β*, *IL-6* and *TNF-α*. *TLR1*, *TLR2*, *TLR3*, *TLR4* (*p* < 0.01), *TLR7* (*p* < 0.05), *TLR8* (*p* < 0.05), and *IL-10* (*p* < 0.01) were positively correlated with *IFN-α*.

*IFN-β* was extremely significant correlated with the relative mRNA expression levels of *TLR5* (*p* < 0.01), *TLR6* (*p* < 0.01), *TLR9* (*p* < 0.01) and *TNF-α* (*p* < 0.01), significantly correlated with *TLR8* (*p* < 0.05). *IFN-β* was negatively correlated with *TLR1*, *TLR4*, *TLR5* (*p* < 0.01), *TLR9* (*p* < 0.01), *IL-1β*, *IL-6* and *TNF-α* (*p* < 0.01). *TLR2*, *TLR3* and *TLR6* (*p* < 0.01), *TLR7*, *TLR8* (*p* < 0.05), *IL-10*, and *IFN-α* were positively correlated with *IFN-β*.

## 4. Discussion

In summary, 10% and 20% stems and leaves of *Panax notoginseng* in diets up-regulated the mRNA expression levels of TLR3, TLR6, TLR7, and TLR8 in the liver of Duzang pigs, along with TRIF, IFN-α, IFN-β, and IL-10. Conversely, they down-regulated the expression of TLR5, TLR9, IL-1β, and TNF-α.

**Figure 7:**
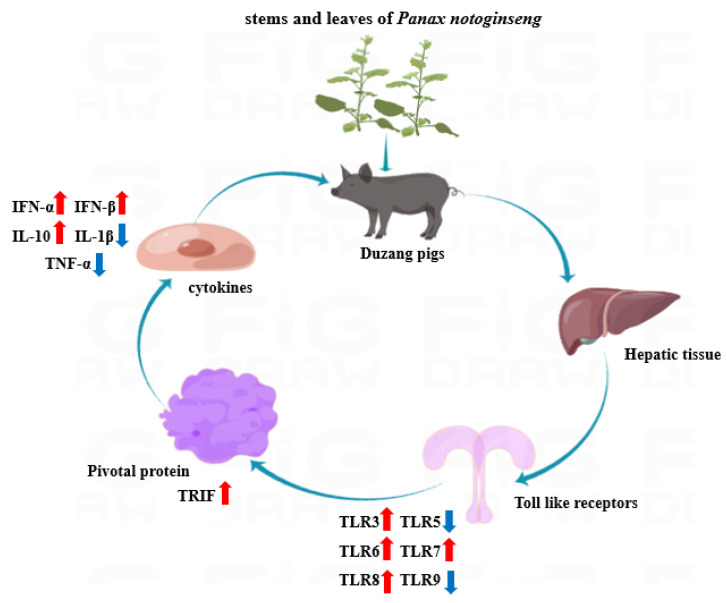
The effect of adding stems and leaves of *Panax notoginseng* on the TLR signaling pathway in the hepatic tissue of Duzang pigs. Note: blue arrows indicate down-regulated mRNA levels and red arrows indicate up-regulated mRNA levels.

Porcine disease resistance is closely associated with multiple genetic, immunological, and physiological mechanisms [15]. Among these, TLRs, the key pattern recognition receptors of innate immunity, recognize pathogens and activate MyD88- and TRIF-dependent immune signaling pathways, thereby triggering a cascade of responses that modulate related immune functions. As shown in Figure 7, in this study, dietary supplementation with varying levels of PNSLs induced differential changes in the mRNA expression levels of TLR family genes in the liver of Duzang pigs. The proteins MyD88 and TRIF, which serve as critical bridging molecules, exhibited corresponding fluctuations in accordance with TLRs expression levels, ultimately leading to dynamic variation in the expression of downstream cytokines.

Crude fiber, an indigestible component in plant-based diets, exerts beneficial effects on swine growth performance despite being non-digestible by porcine enzymes. Substantial evidence indicates that optimal dietary fiber supplementation enhances gut microbiota metabolic activity and improves gastrointestinal motility [16]. PNSLs contain 20.45% crude fiber, suggesting potential for promoting porcine intestinal development through analogous mechanisms. The change in dietary fiber content leads to a significant influence on the main microbial communities that degrade fibers, such as *Firmicutes* and *Actinobacteria* [17]. It will also have an impact on the abundance of *Actinobacteria*, *Bacillota* or *Fibrobacteres*, thereby influencing the production of metabolites [18].

Without containing cellulase, the pig stomach cannot digest the fiber, while the intestinal microorganisms can produce thousands of enzymes [19]. Through fermentation, fibers are converted into substances that are easily absorbed by the host, such as short-chain fatty acids [20]. Interestingly, some short-chain fatty acids can provide energy to intestinal cells, enabling them to function better and promoting the immune function of the intestine. Propionate not only affects liver metabolism, but inhibits the growth of pathogenic bacteria [21]. The intestine can regulate with the liver. The nutrients absorbed by the intestine, toxins, and metabolic products of microorganisms enter the liver through the portal vein. The liver regulates intestinal function by secreting bile acids and immune factors, forming the gut–liver axis [22]. Therefore, it can be seen that this experiment, which explores the effect of the stem and leaves of PN on the mRNA expression level of the TLR signaling pathway genes in the livers of Duzang pigs, may be achieved through the gut–liver axis.

PN polysaccharides exhibit hepatoprotective and immunomodulatory properties. They effectively ameliorate alcohol-induced liver injury by normalizing the aberrant expression of hepatic enzymes. Specifically, PN polysaccharides upregulate alcohol dehydrogenase, the antioxidant enzymes superoxide dismutase and glutathione peroxidase to normal levels, while down-regulating alanine aminotransferase, aspartate aminotransferase, triglycerides, and malondialdehyde, thereby restoring hepatic homeostasis [23]. Furthermore, neutral polysaccharides derived from PN not only suppress the proliferation of hepatocellular carcinoma cell lines, suggesting potential effects on liver cancer treatment [24], but significantly enhance TLR2 protein expression and the secretion of pro- and anti-inflammatory cytokines, including TNF-α, IL-2, IL-10, and IFN-γ, indicating their role in modulating immune responses [25]. PN saponins suppress TLR4 signaling, up-regulate the expression of tight junction proteins (ZO-1 and Claudin-1), enhance intestinal barrier integrity, reduce intestinal permeability, and ameliorate intestinal leakage [26]. Additionally, PN saponins promote the proliferation of primary hepatocytes by increasing the phosphorylation of PI3K, AKT, and mTOR, thereby activating the associated signaling pathways and improving hepatic function [27].

Notably, the feed efficiency of EG I and EG II were increased relative to CG [13,14]. The enhancement may be attributed to synergistic interactions between dietary fiber and bioactive compounds unique to PN, including polysaccharides [28] and saponins [3], which collectively modulate nutrient digestibility and growth performance.

One of the primary KEGG pathways regulated by PN is the TLR signaling pathway [29,30]. The expression levels of *TLR5* and *TLR9* mRNA in CG were significantly higher than that in EG. The expression levels of *TLR3*, *TLR4*, *TLR7* and *TLR8* in EG I were significantly higher than that in CG. Notoginsenosides exert hepatoprotective effects through TLR4-dependent regulation of the gut–liver axis [26]. *TLR4* mRNA expression level of EG I was significantly higher than that of CG and EG II. The results demonstrate that 10% dietary supplementation with PNSLs effectively modulates TLR4-mediated hepatic regulatory mechanisms.

Ginsenoside, the main immunoactive substance of PN, not only inhibits macrophage activation by down-regulating TLR2/4, MyD88, interleukin-1 receptor-associated kinase and transforming growth factor β-activated kinase 1, but also interrupts pathogen-associated molecular pattern signaling through the TLRs/MyD88/NF-κB pathway [31]. MyD88 functions as a crucial adaptor protein that mediates signal transduction between TLRs and the NF-κB pathway. The mRNA expression level of *MyD88* in EG I was significantly higher than that in CG and EG II. The differential mRNA expression between the EG I and EG II may be attributed to the varying supplementation levels of PNSLs.

The study provides evidence that the extract of PN up-regulated the expression levels of *IL-1β* and *TNF-α* in cells [32]. The qPCR revealed that the mRNA expression levels of *IL-1β* and *TNF-α* in EG I were significantly down-regulated compared to the CG. The present study was designed to investigate the effects of dietary supplementation with PNSLs on the hepatic expression levels of *IL-1β* and *TNF-α* in Duzang pigs. In previous studies, inconsistent results could stem from variations in the quality and concentration of PN extract used for treating murine cells, as well as the inherent differences between tissue and cellular responses. PN enhances bacterial clearance in sepsis by modulating adaptive immune responses, including suppression of TNF-α, IL-10, and IL-6 secretion, while concurrently reducing cellular apoptosis and increasing neutrophil counts. Network pharmacology analysis revealed that bioactive compounds in PN modulate the expression of key inflammatory mediators, including TNF-α, IL-6, IL-1β, and IL-10 [29]. The expression level of *IL-6* in EG was not significantly different from that in CG, but the expression level of *IL-10* in EG I was significantly higher than that in EG II and CG.

TLR1 and MyD88 mRNA expression levels were significantly positively correlated, and the former typically formed heterodimers with TLR2 to activate downstream signals through the MyD88-dependent signaling pathway. Upon ligand recognition by the TLR1/TLR2 heterodimer, the complex recruits adaptor proteins containing TIR domains, which subsequently mediate the docking of MyD88 to the receptor assembly [33]. Unlike other TLRs that employ both the MyD88 and TRIF pathway, TLR3 uniquely depends on TRIF pathway for signal transduction after recognizing double-stranded RNA, potentially explaining its specialized role in immunity [34], and TLR3 can recruit tumor necrosis factor receptor-related factor 3 after binding with TRIF. Following the formation of the TLR3-TRIF complex, tumor necrosis factor receptor associated factor 3 is recruited, which subsequently interacts with TRAF family member associated NF-κB activator (TANK) and TANK-binding kinase 1, leading to their phosphorylation and dimerization [35], promoting the production of type I interferons and pro-inflammatory cytokines [36]. Correlation analysis showed that the correlation coefficient of *TLR3* and *TRIF* was 0.456, which was positive, while the correlation coefficient of *TLR3* and *MyD88* was 0, indicating that *TLR3* was completely dependent on TRIF pathway for subsequent expression. *TLR4* activates the immune response through both MyD88 and TRIF dependent signaling pathways [37], and the mRNA expression level is positively correlated with *TRIF* and *MyD88*.

Cytokines are signaling molecules secreted by cells in response to external stimulus, which play pivotal roles in modulating cellular differentiation, proliferation, angiogenesis, and inflammatory responses. Activated TLRs initiate downstream signaling through multiple transcription factors, particularly nuclear factor-κB (NF-κB), leading to the production of diverse cytokines [38], including interferons, tumor necrosis factor family members, and interleukins, function as autocrine, paracrine, and/or endocrine factors that modulate responses in various cell types by binding to their target receptors [39]. TLR9 can promote the release of diverse cytokines through signal transduction pathways [40], including IL-1β and TNF-α, and *TLR9* is positively correlated with both in the research. TLR5 promotes the production of TNF-α by recognizing flagellin in Gram-positive/negative bacteria, and, at the same time, activates NF-κB [41,42] to regulate other cytokines. *TLR5* was positively correlated with *TNF-α*.

## 5. Conclusions

In conclusion, PNSLs with different supplemental levels had regulatory effects on the mRNA expression levels of Toll-like receptors, pivotal proteins and cytokines in hepatic tissue of Duzang pigs to varying degrees. It can be seen that the regulatory mechanism of the PNSLs in TLR signaling pathways needs to be further investigated in order to provide theoretical data for the rational utilization of the PNSLs.

## Figures and Tables

**Table 1:** Nutritional components of PNSLs (air-dried basis). Unit: %.

Raw Material	Dry Matter	Crude Protein	Crude Ash	Crude Fat	Crude Fiber	Calcium	Phosphorus	NFE
PNSLs	94.65	12.45	8.38	2.52	20.45	1.28	2.23	50.85

**Table 2:** Predict mw and pI of TLR1-TLR9 proteins.

	TLR1	TLR2	TLR3	TLR4	TLR5
mw ^1^	90,967.44	89,627.22	103,771.83	96,331.93	97,175.10
pI ^2^	5.87	7.53	7.18	6.10	6.74
	**TLR6**	**TLR7**	**TLR8**	**TLR9**	
mw	91,459.77	120,903.39	118,963.22	115,942.86	
pI	5.97	7.12	6.65	8.96	

mw ^1^ refers to molecular weights. pI ^2^ refers to the theoretical isoelectric points.

**Table 3:** Correlation mRNA expression levels between TLRs and pivotal proteins.

	TLR1	TLR2	TLR3	TLR4	TLR5	TLR6	TLR7	TLR8	TLR9	MyD88	TRIF
TLR1	1.000										
TLR2	−0.317	1.000									
TLR3	0.282	−0.059	1.000								
TLR4	0.408	0.256	0.072	1.000							
TLR5	0.145	−0.528 *	−0.231	−0.332	1.000						
TLR6	−0.422	0.456	0.037	−0.220	−0.695 **	1.000					
TLR7	0.270	0.143	0.395	0.704 **	−0.634 *	0.214	1.000				
TLR8	−0.044	0.431	0.432	0.474	−0.834 **	0.497	0.794 **	1.000			
TLR9	0.451	−0.396	−0.246	0.029	0.727 **	−0.734 **	−0.406	−0.622 *	1.000		
MyD88	0.604 *	−0.495	0.000	0.171	0.506	−0.670 **	−0.150	−0.470	0.875 **	1.000	
TRIF	0.198	0.120	0.456	0.546 *	−0.631 *	0.222	0.821 **	0.735 **	−0.433	−0.182	1.000 ^1^

^1^ r < 0 is negative correlation; r > 0 is positive correlation; |r| < 0.3 is weak correlation; 0.3 < |r| < 0.7 is moderate correlation; 0.7 < |r| < 1.0 is strong correlation. **, at the 0.01 level (two-tailed), the correlation is highly significant. *, at the 0.05 level (two-tailed), the correlation is significant. Same table below.

## Data Availability

The original contributions presented in this study are included in the article. Further inquiries can be directed to the corresponding authors.

## Supplementary Materials

The following supporting information can be downloaded at: https://www.mdpi.com/article/10.3390/genes16070781/s1, Table S1: Composition of basal diet and experimental diet; Table S2: Gene primer sequence and annealing temperature; Table S3: Amino acid composition; Table S4: Correlation between TLRs and expression of cytokine-related genes.

## Abbreviations

The following abbreviations are used in this manuscript:

TLRs	Toll-like receptor
TIR	Toll/interleukin-1 receptor domain
PNSL	*Panax notoginseng* stems and leaves
MyD88	myeloid differentiation factor 88
TRIF	TIR domain-containing adaptor inducing IFN-β
TNF-α	tumor necrosis factor-alpha
IL-1β	interleukin-1 beta
IFN	Interferon
IL	Interleukin
NF-κB	nuclear factor-kappa B
