# Supplementary material for: Effects of Stems and Leaves of Panax notoginseng on mRNA Expression of TLR Signaling Pathway in Hepatic Tissue of Duzang Pigs
Source: Genes (Basel). 2025 Jun 30;16(7):781. doi: 10.3390/genes16070781 (PMC12294736; doi:10.3390/genes16070781)
Supplement: Supplementary file 1 [file genes-16-00781-s001.zip › genes-3697653-supplementary.pdf]

Table S1. Composition of basal diet and experimental diet

Ingredients (%)	CG	EGI	EGII
Corn	72.10	62.50	46.20
Soybean meal	17.40	18.40	18.80
Wheat bran	6.00	0.00	0.00
Soybean oil	0.00	4.60	10.50
Panax notoginseng stems and leaves	0.00	10.00	20.00
Salt	0.50	0.50	0.50
Premix#	4.00	4.00	4.00
Nutritional level			
Digestible energy (MJ/kg)	13.35	13.49	13.62
Crude protein	14.86	14.85	14.86
Lysine	0.67	0.64	0.62
Calcium	0.58	0.59	0.59
Total Phosphorus	0.50	0.52	0.51

#: Provide for pigs: V_A 2200 IU, V_D 700 IU, V_E 14 IU, V_{B6} 1.50 mg, V_{B12} 12 μg, V_{K3} 0.55 mg, folic acid 0.45 mg, V_{B1} 11 mg, biotin 0.05 mg, V_{B2} 2.50 mg, pantothenic acid 10.00 mg, niacin 10 mg, choline chloride 350 mg, selenium 0.25 mg, copper 8 mg, iron 70 mg, zinc 50 mg, manganese 10 mg, iodine 0.35 mg.

Table S2. Gene primer sequence and annealing temperature

gene	primer sequence (5'→3')	annealing temperature
TLR1	F: TTAGGAGATCTTACGGGAA<br>R: ATTTACTGGGTCTGACTGA	58.8°C
TLR2	F: TGCTGCAAGTCAACTCTCT<br>R: CAGCAGGTCACAAGACAGA	52°C
TLR3	F: ATGCTCGAAGGGTGG<br>R: GGGTTGCGTGTTTCC	56.7°C
TLR4	F: ATATGCAGAGGTGAAAGCAC<br>R:GAAGCAGAGATGAAAAGGGG	55°C
TLR5	F: CCACAGGAGTCTTTCGC<br>R: CGGACTTAGTGAGGTGAAT	57.8°C
TLR6	F: ATCACAGCCTCAAGCATTT<br>R: TAGCAGTTGTAAACACCCT	55.8°C
TLR7	F: ATAGGAGCATCACTCCAGCC<br>R: TAACTGCTGCCTTCTGGTGC	62.5°C
TLR8	F: GCTTACGCAAAGACCA<br>R: CAGCTCACAATACTCCTCCC	56°C
TLR9	F: ACAATGACATCATAGCCGAGT<br>R: CAGATCGTTGCGCTAAAGT	59.1°C
MyD88	F: CCTGTCGCTCTCCTAAACG<br>R: AGCCGATAGTGGTCTGTTC	57°C
TRIF	F: TGGGACATCTTAGGGACATG<br>R: CCAGTGGACTCAGGGAATG	58°C

		Attach the table
IL-6	F: GGACGCCTGAAGAAGAT<br>R: TGAACCCAATTGGAAGC	55℃
IL-1β	F: CAGCACTCTCAAGCAGAACAA<br>R: GGCAGAACCATGTACCAACT	58℃
IL-10	F: AAACCACAAGTCCGACAACG<br>R: GAATGCTAGT TCTTCCTCATC	59℃
TNF-α	F: CGCATCGCGTCTCCTACCA<br>R:CTGCCCAGATCAGCAAAGTCCA	55℃
IFN-α	F: CTGTGCCTGGAGATCATCA<br>R: TCCTTCTTCTGAGTCTGTCTTG	60℃
IFN-β	F: CGCTCTCTGATGTGTTTCTCC<br>R: CAAATGCTGCTCCTTTGTTGG	59℃
β-actin	F: CAAGACCTCTACGCCAACAC<br>R: TGGAGCGCGATGATCTT	55℃

Table S3. Amino acid composition

Table S3-1. Amino acid composition of porcine TLR1

Amino acid	Amount	Content（%）
Ala	30	3.8%
Arg	25	3.1%
Asn	51	6.4%
Asp	34	4.3%
Cys	19	2.4%
Gln	40	5.0%
Glu	48	6.0%
Gly	24	3.0%
His	28	3.5%
Ile	56	7.0%
Leu	114	14.3%
Lys	39	4.9%
Met	16	2.0%
Phe	41	5.2%
Pro	26	3.3%
Ser	89	11.2%
Thr	37	4.6%
Trp	10	1.3%
Tyr	25	3.1%
Val	44	5.5%

Table S3-2. Amino acid composition of porcine TLR2

Amino acid	Amount	Content（%）
Ala	37	4.7%
Arg	38	4.8%
Asn	43	5.5%
Asp	38	4.8%
Cys	22	2.8%
Gln	27	3.4%
Glu	46	5.9%
Gly	27	3.4%
His	24	3.1%
Ile	42	5.4%
Leu	119	15.2%
Lys	47	6.0%
Met	12	1.5%
Phe	41	5.2%
Pro	30	3.8%
Ser	82	10.4%
Thr	42	5.4%
Trp	14	1.8%
Tyr	18	2.3%
Val	36	4.6%

Table S3-3. Amino acid composition of porcine TLR3

Amino acid	Amount	Content（%）
Ala	33	3.6%
Arg	32	3.5%
Asn	73	8.1%
Asp	35	3.9%
Cys	16	1.8%
Gln	36	4.0%
Glu	52	5.7%
Gly	32	3.5%
His	31	3.4%
Ile	65	7.2%
Leu	153	16.9%
Lys	54	6.0%
Met	12	1.3%
Phe	54	6.0%
Pro	36	4.0%
Ser	88	9.7%
Thr	41	4.5%
Trp	14	1.5%
Tyr	19	2.1%
Val	29	3.2%

Table S3-4. Amino acid composition of porcine TLR4

Amino acid	Amount	Content（%）
Ala	37	4.4%
Arg	30	3.6%
Asn	56	6.7%
Asp	39	4.6%
Cys	19	2.3%
Gln	45	5.4%
Glu	48	5.7%
Gly	31	3.7%
His	32	3.8%
Ile	47	5.6%
Leu	135	16.1%
Lys	42	5.0%
Met	11	1.3%
Phe	56	6.7%
Pro	31	3.7%
Ser	72	8.6%
Thr	32	3.8%
Trp	9	1.1%
Tyr	21	2.5%
Val	48	5.7%

Table S3-5. Amino acid composition of porcine TLR5

Amino acid	Amount	Content（%）
Ala	38	4.4%
Arg	38	4.4%
Asn	57	6.7%
Asp	47	5.5%
Cys	19	2.2%
Gln	46	5.4%
Glu	32	3.7%
Gly	46	5.4%
His	22	2.6%
Ile	41	4.8%
Leu	148	17.3%
Lys	38	4.4%
Met	13	1.5%
Phe	56	6.5%
Pro	33	3.9%
Ser	73	8.5%
Thr	33	3.9%
Trp	10	1.2%
Tyr	23	2.7%
Val	43	5.0%

Table S3-6. Amino acid composition of porcine TLR6

Amino acid	Amount	Content（%）
Ala	26	3.3%
Arg	25	3.1%
Asn	45	5.7%
Asp	38	4.8%
Cys	23	2.9%
Gln	36	4.5%
Glu	49	6.2%
Gly	23	2.9%
His	26	3.3%
Ile	55	6.9%
Leu	115	14.4%
Lys	47	5.9%
Met	13	1.6%
Phe	46	5.8%
Pro	30	3.8%
Ser	70	8.8%
Thr	46	5.8%
Trp	11	1.4%
Tyr	22	2.8%
Val	50	6.3%

Table S3-7. Amino acid composition of porcine TLR7

Amino acid	Amount	Content（%）
Ala	41	3.9%
Arg	37	3.5%
Asn	83	7.9%
Asp	53	5.0%
Cys	25	2.4%
Gln	44	4.2%
Glu	54	5.1%
Gly	30	2.9%
His	28	2.7%
Ile	53	5.0%
Leu	168	16.0%
Lys	69	6.6%
Met	15	1.4%
Phe	64	6.1%
Pro	47	4.5%
Ser	89	8.5%
Thr	52	5.0%
Trp	15	1.4%
Tyr	32	3.0%
Val	51	4.9%

Table S3-8. Amino acid composition of porcine TLR8

Amino acid	Amount	Content（%）
Ala	41	4.0%
Arg	50	4.9%
Asn	85	8.3%
Asp	52	5.1%
Cys	21	2.0%
Gln	41	4.0%
Glu	50	4.9%
Gly	34	3.3%
His	28	2.7%
Ile	62	6.0%
Leu	164	16.0%
Lys	49	4.8%
Met	11	1.1%
Phe	63	6.1%
Pro	42	4.1%
Ser	87	8.5%
Thr	61	5.9%
Trp	15	1.5%
Tyr	36	3.5%
Val	36	3.5%

Table S3-9. Amino acid composition of porcine TLR9

Amino acid	Amount	Content（%）
Ala	71	6.9%
Arg	75	7.3%
Asn	56	5.4%
Asp	48	4.7%
Cys	27	2.6%
Gln	39	3.8%
Glu	35	3.4%
Gly	54	5.2%
His	41	4.0%
Ile	18	1.7%
Leu	193	18.7%
Lys	26	2.5%
Met	14	1.4%
Phe	49	4.8%
Pro	55	5.3%
Ser	86	8.3%
Thr	47	4.6%
Trp	17	1.7%
Tyr	22	2.1%
Val	57	5.5%

Table S4. Correlation between TLRs and expression of cytokine-related genes

	TLR1	TLR2	TLR3	TLR4	TLR5	TLR6	TLR7	TLR8	TLR9	IL-10	IL-1β	IL-6	TNF-α	IFN-α	IFN-β
TLR1	1.000														
TLR2	-0.317	1.000													
TLR3	0.282	-0.059	1.000												
TLR4	0.408	0.256	0.072	1.000											
TLR5	0.145	-0.528*	-0.231	-0.332	1.000										
TLR6	-0.422	0.456	0.037	-0.220	-0.695**	1.000									
TLR7	0.270	0.143	0.395	0.704**	-0.634*	0.214	1.000								
TLR8	-0.044	0.431	0.432	0.474	-0.834**	0.497	0.794**	1.000							
TLR9	0.451	-0.396	-0.246	0.029	0.727**	-0.734**	-0.406	-0.622*	1.000						
IL-10	0.338	0.060	0.273	0.496	-0.524*	0.148	0.785**	0.659**	-0.356	1.000					
IL-1β	-0.091	-0.504	-0.569*	-0.496	0.467	-0.085	-0.489	-0.496	0.384	-0.355	1.000				
IL-6	-0.133	-0.014	0.064	-0.378	-0.120	0.05 2	-0.126	0.045	-0.165	-0.159	0.150	1.000			
TNF-α	0.419	-0.242	0.060	-0.070	0.640*	-0.719**	-0.484	-0.467	0.791**	-0.475	0.139	0.083	1.000		
IFN-α	0.224	0.322	0.147	0.717**	-0.404	-0.027	0.609*	0.553*	-0.177	0.698**	-0.445	-0.360	-0.272	1.000	
IFN-β	-0.395	0.340	0.351	-0.179	-0.642**	0.813**	0.343	0.520*	-0.848**	0.274	-0.424	-0.049	-0.697**	0.073	1.000
